# S07-1 Does goal orientation relate to changes in sports club participation from adolescence to early adulthood?

**DOI:** 10.1093/eurpub/ckac093.034

**Published:** 2022-08-29

**Authors:** Katja Rinta-Antila, Kevin Gavin, Linda Ooms, Sami Kokko

**Affiliations:** 1 Faculty of Sport and Health Sciences, University of Jyväskylä, Jyvaskyla, Finland; 2 Sport Department, Athlone Institute of Technology, Athlone, Ireland; 3 Mulier Instituut, Utrecht, The Netherlands

**Keywords:** sports club participation, competitive goal, adolescence, early adulthood, longitudinal study

## Abstract

**Background:**

Sports club participation begins to decrease in adolescence. There is a lack of knowledge, how sports club participation changes from adolescence to early adulthood in Finland, and how goal orientation influences on it. Therefore, the aim of this study is to examine if goal orientation is associated to changes in sports club participation during afore mentioned critical years.

**Methods:**

The study design is longitudinal. A sample of 366 (140 boys, 226 girls) adolescents were followed from age 15 (year 2014) to age 19 (year 2018). Sports club participation (yes/no) and goal orientation (no competitive goal, sports for hobby or physical development/regional, national or international success in adolescence/national, international or professional success in adulthood) were measured using questionnaires. In order to study changes in sports club participation, and goal orientation, descriptive statistics were performed. Gender differences were estimated using Chi-squared tests. A binary logistic regression analysis was carried out to examine the association between sports club participation, goal orientation and gender.

**Results:**

By the age 19, 33% of boys and 43% of girls had dropped out from sports club, 45% of boys and 26% of girls had continued participation, and 21% of boys and 31% of girls never participated (p > 0.01). More boys (57%) than girls (31%) had a success in adulthood as a goal, and more girls (48%) than boys (27%) had a success in adolescence as a goal (p > 0.001). Adolescents with success in adulthood as a goal continued participation in sports club more likely than adolescents without competitive goal (OR = 4.81; 95% CI 2.26-10.23). Furthermore, boys were more likely to continue participation than girls (OR = 1.75; 95% CI 1.02-3.01).

**Conclusions:**

The dropout from sports club activities from adolescence to early adulthood is obvious. Especially the adolescents without a competitive goal and girls are in danger to drop out. This indicates that contemporary forms of sports club activities support adolescents with strong competitive orientation. Therefore, there is a need for sports clubs to develop activities suitable for adolescents with less competitive orientation to ensure their continuous participation in sports club.

